# Estimating Blood Pressure during Exercise with a Cuffless Sphygmomanometer

**DOI:** 10.3390/s23177399

**Published:** 2023-08-24

**Authors:** Kenta Hayashi, Yuka Maeda, Takumi Yoshimura, Ming Huang, Toshiyo Tamura

**Affiliations:** 1 Institute of Systems and Information Engineering, University of Tsukuba, Tsukuba 305-8577, Japan; s2120759@s.tsukuba.ac.jp; 2 Department of Medical and Welfare Engineering, Tokyo Metropolitan College of Industrial Technology, Tokyo 116-8523, Japan; t-yoshim@metro-cit.ac.jp; 3 School of Data Science, Nagoya City University, Nagoya 467-8501, Japan; alex-mhuang@ds.nagoya-cu.ac.jp; 4 Future Robotics Organization, Waseda University, Tokyo 169-8050, Japan; t.tamura3@kurenai.waseda.jp

**Keywords:** blood pressure (BP), photoplethysmogram (PPG), skewness signal quality index (SSQI), feature extraction, long short-term memory (LSTM), bidirectional LSTM, exercise

## Abstract

Accurately measuring blood pressure (BP) is essential for maintaining physiological health, which is commonly achieved using cuff-based sphygmomanometers. Several attempts have been made to develop cuffless sphygmomanometers. To increase their accuracy and long-term variability, machine learning methods can be applied for analyzing photoplethysmogram (PPG) signals. Here, we propose a method to estimate the BP during exercise using a cuffless device. The BP estimation process involved preprocessing signals, feature extraction, and machine learning techniques. To ensure the reliability of the signals extracted from the PPG, we employed the skewness signal quality index and the RReliefF algorithm for signal selection. Thereafter, the BP was estimated using the long short-term memory (LSTM)-based neural network. Seventeen young adult males participated in the experiments, undergoing a structured protocol composed of rest, exercise, and recovery for 20 min. Compared to the BP measured using a non-invasive voltage clamp-type continuous sphygmomanometer, that estimated by the proposed method exhibited a mean error of 0.32 ± 7.76 mmHg, which is equivalent to the accuracy of a cuff-based sphygmomanometer per regulatory standards. By enhancing patient comfort and improving healthcare outcomes, the proposed approach can revolutionize BP monitoring in various settings, including clinical, home, and sports environments.

## 1. Introduction

Hypertension diagnosis is a common procedure in clinical practice, and its associated morbidity has doubled over the past decade [1]. Thus, early diagnosis and treatment are critical for preventing hypertension. For blood pressure (BP) monitoring, daily home measurements of BP are preferred over clinical situations owing to the white-coat effect [2,3].

To mitigate the risks of high BP, physicians recommend behavioral and lifestyle changes with dietary control, appropriate nutrition, and quality sleep. Moreover, exercise aids in lowering BP, managing weight, and relieving mental stress. Although BP may be temporarily elevated after exercise, extreme fluctuations may indicate hypertension. Physiologically, systolic blood pressure (SBP) increases during exercise, whereas diastolic blood pressure (DBP) remains relatively stable. Therefore, controlling and monitoring BP during exercise is essential, especially during therapy.

Cuff-based measurement paradigms have dominated ambulatory BP measurement for decades. However, the accurate measurement of BP during exercise is challenging owing to the complexity of modeling the physiological dynamics. Currently, cuffless sphygmomanometers are gaining prominence as they provide adequate accuracy and validity for measuring BP at rested conditions [4,5,6]. Noninvasive optical-based cuffless methods offer various advantages, such as continuous measurements that do not cause disturbance and provide an unconstrained modality to the subjects. However, motion artifacts create challenges for optical-based cuffless sphygmomanometry, and measurements during exercise have not been attempted [7,8]. Moreover, the proposed model must consider rapid alterations in physiological parameters during exercise. 

Various biological applications of machine learning have been studied, such as individualized blood pressure control methods [9] and prediction of hypotension [10]. In particular, machine learning (ML) continuous non-invasive blood pressure (NIBP) monitoring is currently attracting attention in the field of health monitoring due to its various potential benefits, including early prediction of blood pressure [11]. Among them, it has been reported that the MAX86150 module’s sensing and machine learning have resulted in blood pressure estimation accuracy with an error 5.7 ± 5.5 mmHg (±mean ± standard deviation) [12]. Many of these noninvasive optical-based cuffless methods based on machine learning assume resting conditions. This is because changes in vascular resistance and cardiac output associated with exercise are thought to affect the relationship between each parameter obtained from the PPG waveform used in machine learning and blood pressure. In the detection of steep blood pressure fluctuations, which is the target of this study, updating the learning model according to changes in vascular dynamics is an issue. In addition, since body motion artifacts are generated in the PPG signal due to movement, it is necessary to take countermeasures against deterioration of signal quality.

Although a significant increase in systolic BP is a risk factor [13] and needs to be monitored accurately, motion artifacts negatively affect the accuracy of BP measurements. In this study, we proposed a new preprocessing technology and performed BP estimation during exercise.

## 2. Principle

To date, various methods have been developed to estimate BP using cuffless sphygmomanometers, including the photoplethysmogram (PPG)-based cuffless sphygmomanometer that utilizes the PPG signal singularly or coupled with other biosignals, such as the electrocardiogram, phonocardiogram, impedance signal, and ballistocardiogram [14]. An alternative method for estimating blood pressure involves utilizing pulse demodulation with a second derivative signal extracted from the PPG. Pulse demodulation analysis (PDA) is a technique used to evaluate arterial pressure by tracking mechanical events, such as heart contractions and pressure pulse reflections in the central and peripheral arteries. In particular, prior research has identified two major reflection sites in central arteries.

**Figure 1:**
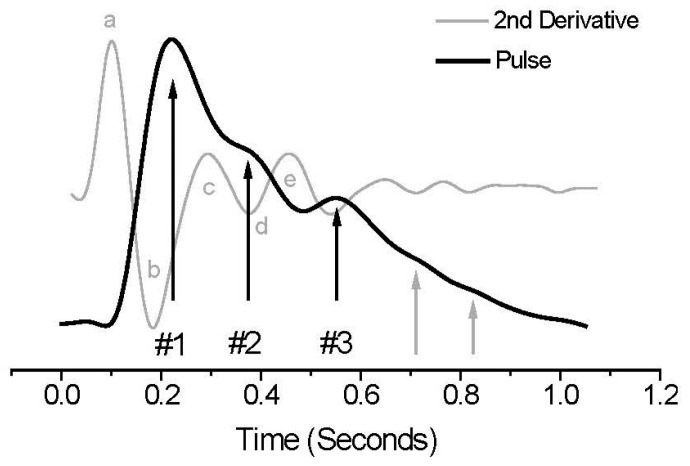
PPG signal and its derivative (gray line).

The pressure waveform obtained by applying the PDA and its second derivatives reveals the typical trend of PPG signals and the characteristics of their second derivatives in Figure 1. The downward-traveling primary pressure pulse (#1) gives rise to the upward-traveling pulses #2 and #3, originating from the renal and iliac reflection sites, respectively, which are impinged upon by pulse #1. The amplitude ratio of the first reflection pulse (#3) to the primary systolic pulse (#1) can be utilized to track changes in the central beat-to-beat SBP [15]. The time difference between the arrival of the first and second reflection pulses (P3) is referred to as *T*_1–3_, which represents the variations in arterial PP. Conventionally, BP is estimated by analyzing the pulse peaks and parameters integrated within the PDA model [15,16,17,18,19].

Lumped parameter models of the cardiovascular system are commonly employed in PDA to simulate the arterial BP waveform and wave propagation, wherein the SBP and DBP are fitted using the resistor impedance and capacitance. Thus, BP can be measured not only by a pressure sensor but also by r by analyzing the PPG waveforms as a reference. Herein, the second derivative of the PPG (SDPPG) signal was analyzed based on the amplitudes of waves a–e, which were produced during the systolic phase of the heart cycle [20] (Figure 1). Moreover, the wave amplitudes were normalized to b/a, c/a, d/a, and e/a. As the SDPPG signal contains information on the aortic compliance and stiffness, which are highly correlated with BP, neural networks and a support vector machine can be employed for numerical analysis of the BP based on the PPG and SDPPG signals.

However, as suggested in previous studies, physiological dynamics during physical exercise differ from that prevailing in the rest state [21,22,23]. For instance, exercise alters the behavior of the baroreflex. It also affects the relationship between the heart rate and the pre-ejection period. Therefore, the BP variations occurring during exercise cannot be accurately predicted using the rest state model. In this study, we assumed that BP regulation strives to stably maintain the BP values during exercise by considering the preceding and subsequent readings of BP in addition to the current measurements. In order to incorporate this assumption, a neural network layer consisting of long short-term memory (LSTM) was employed to capture the informative PPG features over a specific time period for predicting blood pressure. To incorporate this assumption, a neural network layer of long short-term memory (LSTM) was used to associate the informative PPG features over three different timespans for BP prediction. The response of PPG parameters such as heart rate and waveform shape to exercise is rapid. In contrast, changes in blood pressure are relatively gradual. PPG parameters should be considered not only unique features but also the context of the time series. Therefore, we selected LSTM because it is the only one capable of capturing features of long-term time series data for estimating blood pressure fluctuations in this study. We expect that the combined approach of physical modeling and data-driven approaches will provide an accurate BP measurement during exercise, especially in data-scarce scenarios.

## 3. Data Collection and Signal Processing

**Figure 2:**
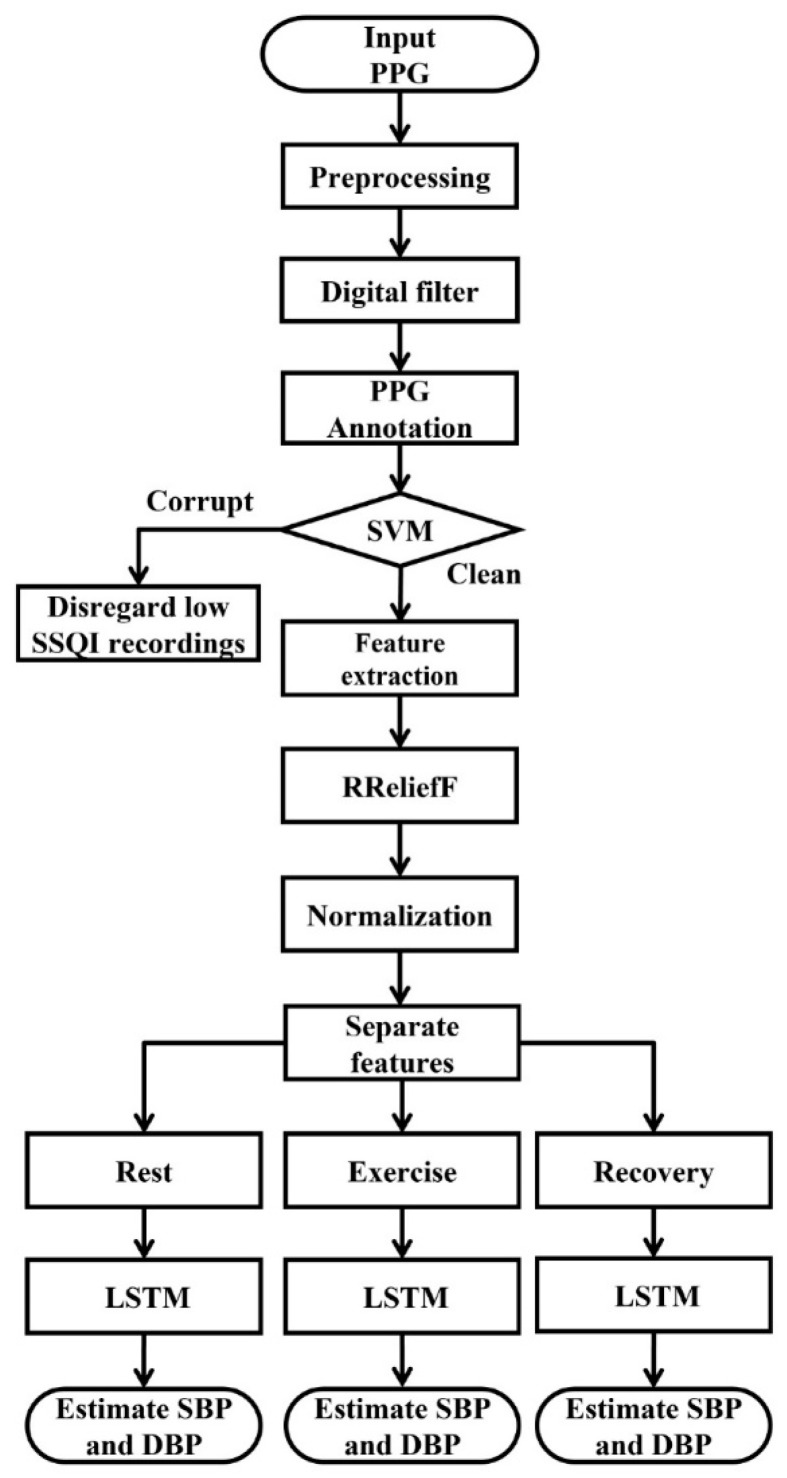
Signal processing for BP estimation.

In this study, the proposed BP estimation method involved signal preprocessing, feature extraction, and BP estimation; the processing procedure is illustrated as a flowchart in Figure 2. 

### 3.1. Experimental Method

Data were collected from 20 young males (age: 19.7 ± 1.22 years; range: 18–23 years; BMI: 21.62 ± 2.83; range: 16.23–29.06). The age range of the participants was narrow because of the relatively high safety involved with young participants during long-term exercise. 

For reference, the SBP, DBP, and PPG were measured sequentially using a voltage clamp-type continuous BP monitor (CNAP, CNSystem, Graz, Austria). CNAP is based on the vascular unloading technique. It is the basic principle for detecting blood volume changes in the finger and transforming plethysmographic signals into continuous blood pressure information. Blood pressure can then be calculated beat-to-beat, after calibration with the built-in standard oscillometric measurement (NBP) of the monitor. From these protocols, the gap between intermittent NBP and continuous invasive blood pressure is bridged. Before and after the experiments, a cuff-based sphygmomanometer (HEM-7511T, Omron, Kyoto, Japan) was used to measure the SBP and DBP for further reference.

The experiment consisted of three parts. After sitting on an ergometer (TE3PLUS-70, Showa Denki Group, Osaka, Japan), the subjects initially rested for 20 min, followed by exercise with the ergometer for another 20 min at 80 W and 50 rpm; after this, the subjects were allowed to recover for 20 min. Due to the challenges related to measurement, peri-exercise has received less investigation as an effective method for inducing significant variations in blood pressure. To the best of our knowledge, this is one of the few studies dedicated to cuffless BP estimation during exercise [24,25]. The experiment was approved by the Ethics Committee of the Tokyo Metropolitan College of Industry Engineering (SanGisenkanAra #578) and written informed consent was obtained from each participant prior to the experiment. The strength and intensity of the exercise followed the Karvonen formula, defined as the maximum and resting heart rates with the desired training intensity to obtain the target heart rate:

Target Heart Rate = [(max HR − resting HR) × % intensity] + resting HR benchmark,

where max HR was defined as 220 and age % intensity was set as 0.7 based on a previous report [26].

### 3.2. Skewness Signal Quality Index

**Figure 3:**
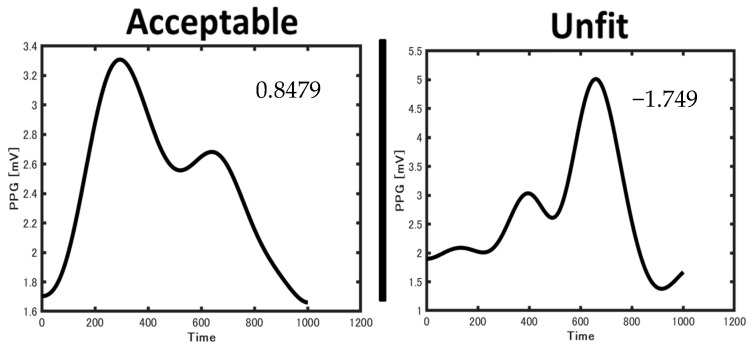
Examples of acceptable and unfit PPG waveform.

During the preprocessing, a skewness signal quality index (SSQI) was applied to each PPG signal. As motion artifacts can affect the PPG signals during exercise, the signal quality must be appropriately annotated. The increased skewness of the PPG signals revealed a detailed morphology of the pulse waveform [27]. Therefore, skewness (i.e., the optimal SQI) can potentially be used to improve the diagnosis and monitoring of abnormalities such as hypertension. The optimal SQI for PPG-based technologies forms the first step toward BP estimation. We focused on improving the accuracy of diagnoses and the quality of care by applying SSQI to acquire high-quality signals in various conditions (e.g., resting, exercise, and recovery). The annotation of the entire signal was based on the most dominant beat wave quality within the signal, which enabled clear classification of the groups into acceptable and unfit signals for estimating the SBP and DBP exhibited in Figure 3. 

Skewness is a measure of the symmetry (or lack thereof) of a probability distribution, defined as follows:

(1) $$SSQI=\frac{1}{N}\sum_{i=1}^{N}{\left[x_{i}-\frac{\hat{\mu_{x}}}{\sigma }\right]}^{3},$$

where $\hat{\mu_{x}}$ and *σ* denote the empirical estimate of the mean and standard deviation of *x_i_*, respectively, and *N* indicates the number of samples in the PPG signal. The *SSQI*s were calculated for each PPG signal.

The distribution of each SQI within a given subset exhibited significant variability, making a simple fixed threshold inadequate for optimal classification. A linear support vector machine (SVM) was utilized as the classifier, and each annotator annotated the PPG signals based on the most dominant PPG signal. Our primary focus was to determine the optimal SQI for which a simple classifier with a fixed threshold would be satisfactory. However, there were variations in the distribution of each SQI, making a simple fixed threshold unsuitable as an optimal classifier. Consequently, the robust SQIs were determined for each phase. 

### 3.3. Feature Extraction

To extract information from the signal, all signals were normalized using the *Z*-score techniques and obtaining the amplitude-limited data.

(2) $$Z_{score}=\sum_{i=1}^{n}[(y_{i}-M)/SD],$$

where *M* and *SD*, respectively, denote the mean and standard deviation of the feature value *y_i_*, and n indicates the number of samples. 

**Figure 4:**
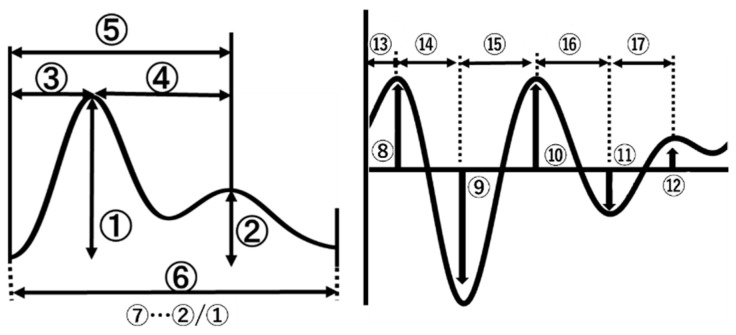
Features of PPG signals and its derivatives. Details of numbers in the figure are presented in Table 1.

The 17 features presented in Figure 4 and Table 1 were selected from a single PPG signal and its derivatives. Subsequently, the feature selection was performed using the RReliefF algorithm [28,29], which is a feature selection algorithm that randomly selects instances and adjusts the weights of the respective elements depending on the nearest neighbor. The built-in functions of MATLAB were used in this study. 

### 3.4. Machine Learning Model: Long Short-Term Memory

**Figure 5:**
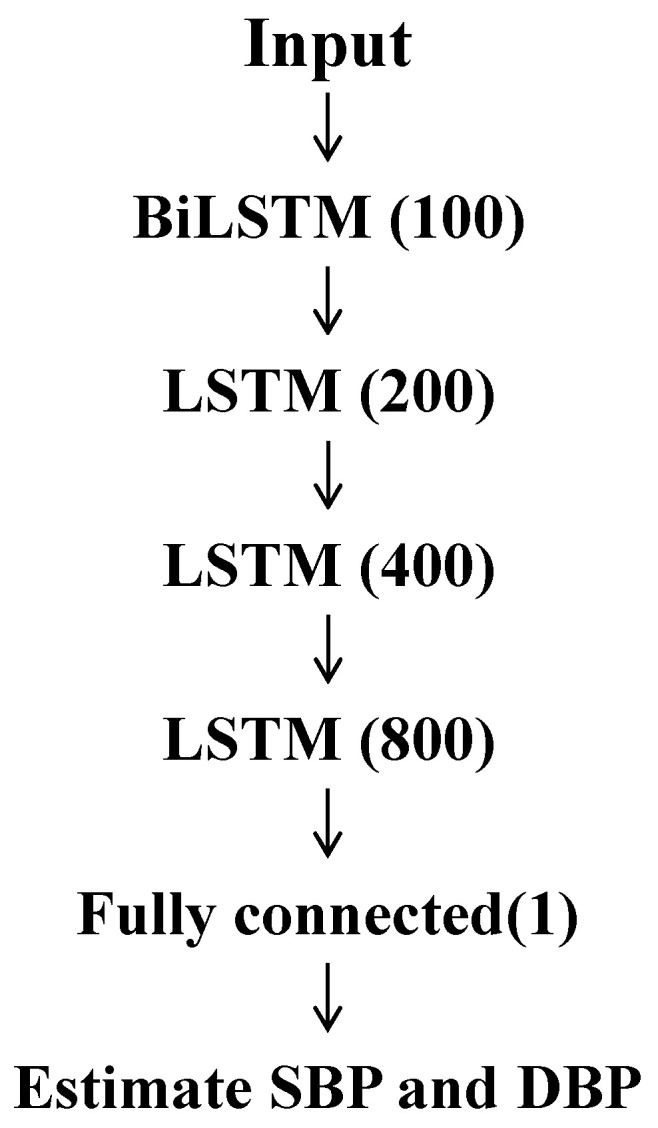
Structure of LSTM.

After feature extraction, the feature matrix was trained using machine learning algorithms. The analysis was performed in three phases: rest, exercise, and recovery. The LSTM layer, designed for sequence learning, proves effective in estimating BP [30]. In this study, we employed it as the primary component of our learning model. To validate the fundamental assumption, the LSTM comprised a single-layer bidirectional LSTM (BiLSTM), followed by three layers of LSTM, a fully connected (FC) layer, and a regression layer as the output. In particular, the BiLSTM was used in the first layer to capture information from the accepted input signal. All three phases used the same model with an architecture, initiating with a single layer of BiLSTM, consisting of 100 hidden units, followed by three LSTM layers with hidden sizes of 200, 400, and 800 units, respectively. The mini-batch size was 256 with 850 epochs and an initial learning rate of 0.004. The details of the architecture are displayed in Figure 5. In each phase, 70%, 15%, and 15% of data were used as training, test, and evaluation data, respectively. 

### 3.5. Error Metrics

To measure the error from the models used in the experiments, mean absolute error (*MAE*), root mean square error (*RMSE*), and standard deviation (STD) were evaluated from the experiments.

Two criteria were used to evaluate the performance of the LSTM algorithms in estimating the BP. *X_p_* represents the predicted data, *X* denotes the ground truth data, and *N* indicates the number of samples. 

*MAE*: The absolute error denotes the predicted error, whereas the *MAE* represents the mean of all absolute errors.

(3) $$MAE=\frac{1}{N}\sum_{N}\left|X_{p}-X\right|.$$

*ME*: *ME* calculates the squared sum of the errors, representing the expected value of the squared-error loss.

(4) $$ME=\frac{\sum \left|X_{p}-X\right|}{N}.$$

*RMSE*: The *RMSE* denotes the standard deviation of the residuals (prediction error).

(5) $$RMSE=\sqrt{\frac{\sum {\left|X_{p}-X\right|}^{2}}{N}}.$$

Correlation coefficient (*R*):

(6) $$R=\sqrt{1-\frac{MSE\left(Model\right)}{MSE\left(Standard\right)},}$$

$$where\;MSE=\frac{\sum {\left\lceil X-meanX\right\rceil }^{2}}{N}.$$

## 4. Results

The present experiment included 20 participants; however, the BP values for three participants could not be measured by the continuous sphygmomanometer. Thus, the dataset for analysis contained BP data from 17 participants.

### 4.1. Preprocessing

**Figure 6:**
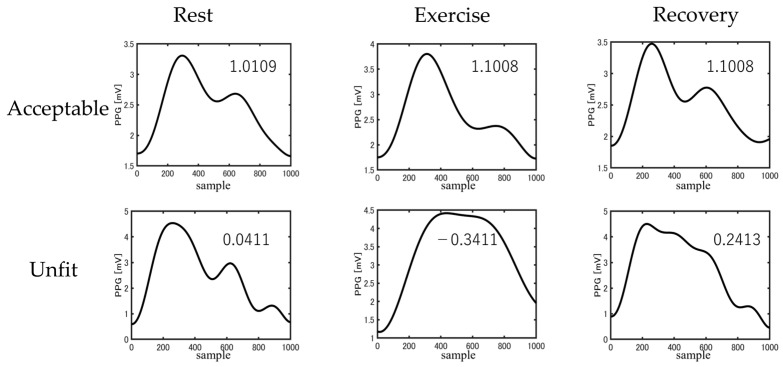
Difference in acceptable and unfit waveforms between three phases.

The typical examples of PPG signals with acceptable and unsuitable waveforms are presented in Figure 6. Although the second peak was clearly observed in the PPG waveforms during rest and recovery, it exhibited a low amplitude during exercise. The rejection ratios of the PPG signals after *SSQI* analysis are listed in Table 2, wherein the rejection rates were 23.9% at rest, 69.0% during exercise, and 16.1% during recovery.

### 4.2. Feature Extraction

The SBP and DBP scores are summarized in Table 3. As the negative scores were relatively high during exercise, the features with negative scores were eliminated from the LSTM input gate.

### 4.3. BP Estimation

**Figure 7:**
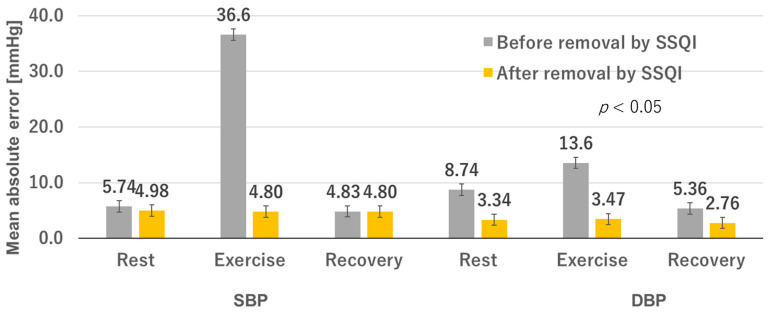
BP estimation before and after SSQI processing.

The estimation errors before and after SSQI processing are plotted in Figure 7, and the predicted SBP and DBP values are summarized in Table 4. The extraction of the chaotic features from vital signals improved the accuracy of the BP estimation results. Notably, the BP estimation results were evaluated using ISO standards.

**Figure 8:**
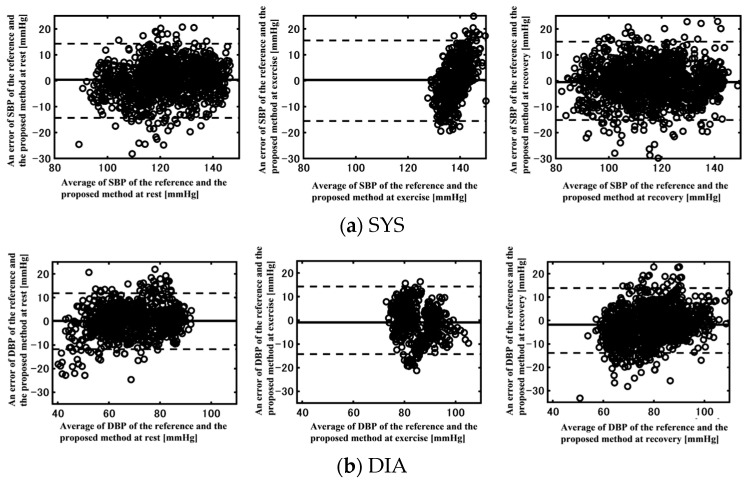
Bland–Altman plots of reference and estimated (**a**) SYS and (**b**) DIA BPs at rest, exercise, and recovery.

The Bland–Altman plots are visualized in Figure 8; all data were statistically acceptable within the 95% confidence limit.

## 5. Discussion

The BP estimation was accurate with noise reduction and feature extraction machine learning techniques. 

Motion artifacts during exercise affected the PPG measurement. The *SSQI* exhibited relatively high signal quality indices (SQIs). In particular, a higher rejection rate for high-error signals and a lower *MAE* were achieved. As such, daily BP monitoring requires considerable care when attaching a PPG sensor. 

The heightened cardiac pulse during exercise expands the arterial wall which then decreases the thickness of the arterial wall depending on arterial stiffness. We assumed that the arterial wall is incompressible, isotropic, and exhibits no strain in the axial direction. Accordingly, the elasticity *E* can be expressed as follows:

(7) $$E=\frac{3}{8}(1+\frac{{2r}_{0}}{h_{0}})\frac{∆p}{∆h/h_{0}},$$

where *r*_0_ denotes the end-diastolic radius, h_0_ indicates wall thickness, Δ*p* represents pulse pressure, and Δ*h* denotes the variation in thickness per cardiac cycle [31,32].

**Figure 9:**
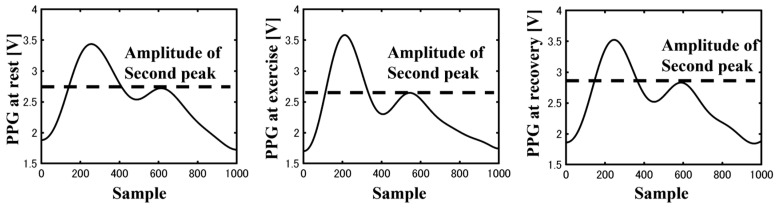
Second peak of PPG amplitude at rest, exercise, and recovery.

The second peak corresponded to the lower elasticity, which altered the pulse pressure and wall thickness. During exercise, the first peak corresponds to a large pulse pressure and thinner wall thickness, which bears a lower elasticity. As the second peak corresponds to the reflected wave, the second peak is typically lower owing to the lower elasticity (Figure 9). A typical example of a PPG waveform in three distinct phases is presented in Figure 9 to clearly illustrate the differences across the rest, exercise, and recovery phases.

**Figure 10:**
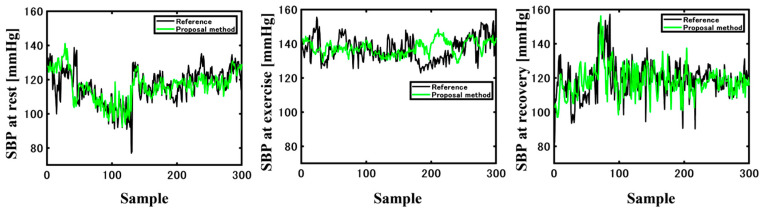
Reference BP (black line) and estimated BP (green line) at rest, exercise, and recovery.

The Bland–Altman plot of SBP error indicated a systemic error. Although the range of the systolic BP was extremely narrow, the error between the reference and estimated BPs was linear. The time course of BP at three distinct epochs is depicted in Figure 10, during which the trend of the estimated systolic BP closely followed the reference BP at the rest and recovery phases. However, during exercise, the SBP did not progress appropriately due to the high rejection rate. As the LSTM algorithm is specialized on time series features, an exceedingly high rejection rate may induce large variations instead of slight fluctuations.

Automated sphygmomanometers, which are currently widely used, are compliant with resting state (ISO 81060-2:2018, Non-invasive sphygmomanometers—Part 2: Clinical investigation of automated measurement type) and blood pressure variation (ISO DIS 81060-3.2, Non-Invasive Sphygmomanometers—Part 3: Clinical Investigation Of Continuous Automated Measurement Type).

According to the above standards, the resting accuracy of automated sphygmomanometers is defined as within ±5 ± 8 mmHg (±mean ± standard deviation), and the blood pressure estimation accuracy required for cuffless sphygmomanometers is also considered to meet this standard.

The device used as a reference in this study (CNAP, CNSystem, Austria) calibrates the blood pressure curve using NBP approved by ISO81060-2 (NBP), and the accuracy of continuous blood pressure has been confirmed.

The errors of the proposed method for systolic blood pressure are 0.50 ± +/− 7.19 mmHg, 0.32 ± 7.76 mmHg, and −0.48 ± 7.55 mmHg (±mean ± standard deviation) for resting, exercise, and recovery periods, respectively; for diastolic blood pressure, 0.39 ± 5.48 mmHg, −0.91 ± 7.15 mmHg, and −1.77 ± 6.94 mmHg (±mean ± standard deviation) at rest, during exercise, and during recovery, respectively. The accuracy was confirmed to follow the accuracy set by ISO standards.

Although the accuracy of the proposed method meets the accuracy of a cuff-type automated sphygmomanometer, there are still issues to be addressed regarding the clinical application of the proposed method. The small number of subjects is one of them. Since the waveform shape used in this study varies with gender and age, it is necessary to verify the method in a wide subject population in the future. In particular, when applied to detect blood pressure fluctuations in diseased groups, it is possible that a change trend different from that of the young adults targeted in this study may occur.

## 6. Conclusions

In summary, we proposed a novel waveform-based LSTM model for continuous blood pressure estimation using PPG waveforms. The proposed model extracted the essential features and captured the temporal variations, yielding BP measurement accuracies that satisfy the existing regulatory standards. The present study addresses the need for noninvasive and accurate blood pressure monitoring during exercise, providing a potential solution for improving patient comfort and healthcare outcomes.

The research demonstrates the feasibility of utilizing machine learning algorithms to analyze PPG signals for blood pressure estimation, demonstrating the effectiveness of the proposed model. The proposed method will aid in the development of cuffless blood pressure monitoring devices, offering a noninvasive and accurate alternative to traditional cuff-based sphygmomanometers.

Note that the limitations of this research include the small sample size and the need for validation in larger cohorts to ensure generalizability and reliability of the proposed model. Thus, in the future, research should focus on refining the model and exploring its performance in diverse populations as well as investigating its integration into wearable devices for real-time blood pressure monitoring. 

## Figures and Tables

**Table 1:** Features of PPG signals.

PPG Signal		
Systolic peak	1	The amplitude of first peak from PPG waveform
Diastolic peak	2	The amplitude of first peak from PPG waveform
Systolic peak time	3	The time interval from the foot of the waveform to the systolic peak (‘t1’)
∆T	4	The time interval from systolic peak time to diastolic peak time
Diastolic peak time	5	The time interval from systolic peak time to diastolic peak time
Pulse interval	6	The distance between the beginning and the end of the PPG waveform
Augmentation index	7	The ratio of diastolic peak amplitude and systolic peak amplitude
Second derivative PPG signal		
Peak a	8	The first maximum peak from the second derivative of the PPG waveform
b	9	The first minimum peak from the second derivative of the PPG waveform
c	10	The second maximum peak from the second derivative of the PPG waveform
d	11	The second minimum peak from the second derivative of the PPG waveform
e	12	The third maximum peak from the second derivative of the PPG waveform
Ta	13	The time interval from the foot of the PPG waveform to the time at which first peal of second derivative occurred
Tb-a	14	The time interval from first maximum peak to first minimum peak
Tb-c	15	The time interval from first minimum peak to second maximum peak
Tc-d	16	The time interval from second maximum peak to second minimum peak
Td-e	17	The time interval from second minimum peak to third maximum peak

**Table 2:** Rejection rate of PPG signal in each phase.

	Rest	Exercise	Recovery
Before processing	14,290	21,174	17,193
After processing	10,878	6566	14,419
Rejection rate [%]	23.88	68.99	16.13

**Table 3:** Feature score at various phases.

	FeatureScore	①	②	③	④	⑤	⑥	⑦	⑧	⑨	⑩	⑪	⑫	⑬	⑭	⑮	⑯	⑰
Rest	SBP	0.0084	0.0066	0.0012	0.0025	0.0020	0.0017	0.0047	0.0013	0.0011	0.0017	0.0032	0.0047	−0.0001	0.0004	−0.0015	−0.0001	0.0014
	DBP	0.0070	0.0065	0.0016	0.0034	0.0031	0.0032	0.0050	0.0036	0.0011	0.0018	0.0029	0.0049	−0.0001	0.0016	−0.0008	0.0001	0.0022
Exercise	SBP	0.0309	0.0203	0.0088	0.0152	0.0164	0.0218	0.0156	0.0202	0.0226	0.0269	0.0288	0.0275	−0.0012	0.0117	0.0100	0.0102	0.0148
	DBP	0.0273	0.0202	0.0081	0.0162	0.0175	0.0246	0.0168	0.0199	0.0179	0.0269	0.0284	0.0303	−0.0008	0.0125	0.0106	0.0125	0.0174
Recovery	SBP	0.0101	0.0076	0.0003	0.0027	0.0030	0.0001	0.0061	0.0022	0.0039	0.0079	0.0057	0.0075	−0.0004	−0.0008	−0.0009	−0.0003	0.0009
	DBP	0.0092	0.0080	0.0002	0.0026	0.0026	0.0005	0.0057	0.0033	0.0030	0.0081	0.0061	0.0064	−0.0003	−0.0003	−0.0003	0.0002	0.0012

**Table 4:** BP statistics.

	SBP			DBP		
	*MAE*	*ME*	*SD*	*MAE*	*ME*	*SD*
Rest	4.98	0.50	7.19	3.34	0.39	5.48
Exercise	4.80	0.32	7.76	3.47	–0.91	7.15
Recovery	4.80	–0.48	7.55	2.76	–1.77	6.94

## Data Availability

Data sharing not applicable.

## References

[1] NCD Risk Factor Collaboration (NCD-RisC) (2021). Worldwide trends in hypertension prevalence and progress in treatment and control from 1990 to 2019: A pooled analysis of 1201 population-representative studies with 104 million participants. Lancet.

[2] George J., MacDonald T. (2015). Home Blood Pressure Monitoring. Eur. Cardiol. Rev..

[3] Asayama K., Thijs L., Brguljan-Hitij J., Niiranen T.J., Hozawa A., Boggia J., Aparicio L.S., Hara A., Johansson J.K., Ohkubo T. (2014). Risk Stratification by Self-Measured Home Blood Pressure across Categories of Conventional Blood Pressure: A Participant-Level Meta-Analysis. PLOS Med..

[4] Tamura T. (2021). Cuffless Blood Pressure Monitors: Principles, Standards and Approval for Medical Use. IEICE Trans. Commun..

[5] Mukkamala R., Stergiou G.S., Avolio A.P. (2022). Cuffless Blood Pressure Measurement. Annu. Rev. Biomed. Eng..

[6] Almeida T.P., Cortés M., Perruchoud D., Alexandre J., Vermare P., Sola J., Shah J., Marques L., Pellaton C. (2023). Aktiia cuffless blood pressure monitor yields equivalent daytime blood pressure measurements compared to a 24-h ambulatory blood pressure monitor: Preliminary results from a prospective single-center study. Hypertens. Res..

[7] Stergiou G.S., Mukkamala R., Avolio A., Kyriakoulis K.G., Mieke S., Murray A., Parati G., Schutte A.E., Sharman J.E., Asmar R. (2022). Cuffless blood pressure measuring devices: Review and statement by the European Society of Hypertension Working Group on Blood Pressure Monitoring and Cardiovascular Variability. J. Hypertens..

[8] Mukkamala R., Shroff S.G., Landry C., Kyriakoulis K.G., Avolio A.P., Stergiou G.S. (2023). The Microsoft Research Aurora Project: Important Findings on Cuffless Blood Pressure Measurement. Hypertension.

[9] Evangelos K., Erica S., Marc A.S., Rohan K. (2022). Individualising intensive systolic blood pressure reduction in hypertension using computational trial phenomaps and machine learning: A post-hoc analysis of randomised clinical trials. Lancet Digit. Health.

[10] Hatib F., Jian Z., Buddi S., Lee C., Settels J., Sibert K., Rinehart J., Cannesson M. (2018). Machine-learning Algorithm to Predict Hypotension Based on High-fidelity Arterial Pressure Waveform Analysis. Anesthesiology.

[11] Ismail S.N.A., Nayan N.A., Jaafar R., May Z. (2022). Recent Advances in Non-Invasive Blood Pressure Monitoring and Prediction Using a Machine Learning Approach. Sensors.

[12] Nidigattu G.R., Mattela G., Jana S. Non-invasive modeling of heart rate and blood pressure from a photoplethysmography by using machine learning techniques. Proceedings of the International Conference on COMmunication Systems & NETworkS (COMSNETS).

[13] Kurl S., Laukkanen J., Rauramaa R., Lakka T., Sivenius J., Salonen J. (2001). Systolic Blood Pressure Response to Exercise Stress Test and Risk of Stroke. Stroke.

[14] Elgendi M., Fletcher R., Liang Y., Howard N., Lovell N.H., Abbott D., Lim K., Ward R. (2019). The use of photoplethysmography for assessing hypertension. NPJ Digit. Med..

[15] Baruch M.C., Warburton D.E., Bredin S.S., Cote A., Gerdt D.W., Adkins C.M. (2011). Pulse Decomposition Analysis of the digital arterial pulse during hemorrhage simulation. Nonlinear Biomed. Phys..

[16] Elgendi M. (2012). On the Analysis of Fingertip Photoplethysmogram Signals. Curr. Cardiol. Rev..

[17] Epstein S., Willemet M., Chowienczyk P.J., Alastruey J. (2015). Reducing the number of parameters in 1D arterial blood flow modeling: Less is more for patient-specific simulations. Am. J. Physiol. Circ. Physiol..

[18] Shin H., Min S.D. (2017). Feasibility study for the non-invasive blood pressure estimation based on ppg morphology: Normotensive subject study. Biomed. Eng. Online.

[19] Gratz I., Deal E., Spitz F., Baruch M., Allen I.E., Seaman J.E., Pukenas E., Jean S. (2017). Continuous Non-invasive finger cuff CareTaker® comparable to invasive intra-arterial pressure in patients undergoing major intra-abdominal surgery. BMC Anesthesiol..

[20] Liu M., Po L.-M., Fu H. (2017). Cuffless Blood Pressure Estimation Based on Photoplethysmography Signal and Its Second Derivative. Int. J. Comput. Theory Eng..

[21] Liu Q., Yan B.P., Yu C.-M., Zhang Y.-T., Poon C.C.Y. (2014). Attenuation of Systolic Blood Pressure and Pulse Transit Time Hysteresis During Exercise and Recovery in Cardiovascular Patients. IEEE Trans. Biomed. Eng..

[22] Miki K., Yoshimoto M. (2018). Exercise-Induced Modulation of Baroreflex Control of Sympathetic Nerve Activity. Front. Neurosci..

[23] Pilz N., Andreas P., Tomas L.B. (2023). The pre-ejection period is a highly stress dependent parameter of paramount importance for pulse-wave-velocity based applications. Front. Cardiovasc. Med..

[24] Esmaili A., Mohammad K., Mahdi S. (2017). Nonlinear Cuffless Blood Pressure Estimation of Healthy Subjects Using Pulse Transit Time and Arrival Time. IEEE Trans. Instrum. Meas..

[25] Sun S., Bezemer R., Long X., Muehlsteff J., Aarts R.M. (2016). Systolic blood pressure estimation using PPG and ECG during physical exercise. Physiol. Meas..

[26] Yabe H., Kono K., Onoyama A., Kiyota A., Moriyama Y., Okada K., Kasuga H., Masuda A. (2021). Predicting a target exercise heart rate that reflects the anaerobic threshold in nonbeta-blocked hemodialysis patients: The Karvonen and heart rate reserve formulas. Ther. Apher. Dial..

[27] Elgendi M. (2016). Optimal Signal Quality Index for Photoplethysmogram Signals. Bioengineering.

[28] Shuzan N.I., Chowdhury M.H., Chowdhury M.E.H., Murugappan M., Hoque Bhuiyan E., Ayari M.A., Khandakar A. (2023). Machine Learning-Based Respiration Rate and Blood Oxygen Saturation Estimation Using Photoplethysmogram Signals. Bioengineering.

[29] Chowdhury M.H., Shuzan N.I., Chowdhury M.E., Mahbub Z.B., Uddin M.M., Khandakar A., Reaz M.B.I. (2020). Estimating Blood Pressure from the Photoplethysmogram Signal and Demographic Features Using Machine Learning Techniques. Sensors.

[30] Li Y.-H., Harfiya L.N., Purwandari K., Lin Y.-D. (2020). Real-Time Cuffless Continuous Blood Pressure Estimation Using Deep Learning Model. Sensors.

[31] Patel D.J., Janicki J.S., Vaishnav R.N., Young J.T. (1973). Dynamic Anisotropic Viscoelastic Properties of the Aorta in Living Dogs. Circ. Res..

[32] Miyachi Y., Arakawa M., Kanai H. (2018). Accuracy improvement in measurement of arterial wall elasticity by applying pulse inversion to phased-tracking method. Jpn. J. Appl. Phys..

